# Lumbar multifidus muscle morphology is associated with low back-related pain duration, disability, and leg pain: A cross-sectional study in secondary care

**DOI:** 10.1371/journal.pone.0285993

**Published:** 2023-06-02

**Authors:** Jeffrey R. Cooley, Per Kjaer, Tue S. Jensen, Angela Jacques, Jean Theroux, Jeffrey J. Hebert

**Affiliations:** 1 College of Science, Health, Engineering and Education, Murdoch University, Murdoch, Western Australia, Australia; 2 Department of Sports Science and Clinical Biomechanics, University of Southern Denmark, Odense M, Denmark; 3 Health Sciences Research Centre, UCL University College, Odense M, Denmark; 4 Department of Diagnostic Imaging, Regional Hospital Silkeborg, Silkeborg, Denmark; 5 Spine Centre of Southern Denmark, Middelfart, Denmark; 6 Chiropractic Knowledge Hub, Odense M, Denmark; 7 Institute for Health Research, University of Notre Dame Australia, Fremantle, Western Australia, Australia; 8 Faculty of Kinesiology, University of New Brunswick, Fredericton, New Brunswick, Canada; Universidade de Sorocaba, BRAZIL

## Abstract

**Background:**

Systematic reviews and studies exploring associations between morphologic change of paraspinal muscles and low back pain or related outcomes such as disability, radiculopathy, and physical workload, have reported conflicting results. This study explores the associations between lumbar multifidus muscle quality and clinical outcomes relating to low back pain.

**Methods:**

Cross-sectional study of spinal clinic outpatients presenting with a primary complaint of low back and/or leg symptoms. Univariable and multivariable regression models were used to investigate associations between MRI-based multifidus muscle cross-sectional area at L4 and L5 and clinical outcomes for low back pain, leg pain, disability, restricted motion, and strenuous nature of work. Results were reported with β-coefficients, odds ratios (OR), or incidence rate ratios (IRR) and their corresponding 95% confidence intervals, based on a 10% difference in muscle quality for each clinical variable. Multivariable analyses were adjusted for age, sex, and BMI.

**Results:**

875 patients [487 females; mean (SD) age: 43.6 (10.2) years] were included. In the multivariable analyses, muscle quality was significantly associated with disability (0–23 scale) [β: -0.74, 95% CI: -1.14, -0.34], leg pain intensity (0–10 scale) [β: -0.25, 95% CI: -0.46, -0.03], and current pain duration of more than 12 months [OR: 1.27, 95% CI: 1.03, 1.55]. No associations were found for low back pain intensity, morning stiffness, painful active range of motion, or work nature.

**Conclusions:**

Patients with higher lumbar multifidus muscle quality reported lower levels of low back pain-related disability and leg pain intensity, indicating that muscle quality may play a role in the etiology of lumbar spine disorders. However, the clinical importance of these associations is uncertain due to the low magnitude of identified associations. Future longitudinal studies are needed to understand the effect of lumbar multifidus muscle quality on lumbar-related pain and disability.

## Introduction

Despite intensive research efforts to enhance our understanding of the causes and management of low back pain, this disorder has become one of the ten most important drivers increasing the global disease burden. It remains ranked 1^st^ among causes of years lived with disability (YLD), is responsible for 7.4% of total global YLD, is found in all age groups from 5 years and older, and in 2019 had a reported overall prevalence rate/100,000 population of 6972.5 [1].

As low back pain due to a specific pathology is rare (< 10%), most patients are classified as experiencing either non-specific low back pain (~90–95%) or, less frequently, low back pain with an associated radicular syndrome (~5–10%) [2, 3]. In their 2001 review of low back pain, Deyo and Weinstein discussed all the factors currently considered to play a key role in the aetiology and management of low back pain [4]. At that time, paraspinal muscle morphology was not mentioned as a risk factor for specific or non-specific back pain. Since then, there has been a growing focus on spinal function, specifically looking at the intrinsic paraspinal musculature and the role of the lumbar multifidi muscles in the cause, progression, and outcomes of low back pain [5].

The lumbar multifidi are complex structures that provide a unique contribution to lumbar spine stability [6, 7], accounting for approximately 2/3^rd^ of the stability at the L4/L5 segment [8]. Spinal instability is a theorized mechanism of low back pain used to justify therapies from exercise to surgical fusion [9–11], and while a systematic review by Fortin et al. [12] reported that the preponderance of evidence supports an association between low back pain and morphologic change of paraspinal muscles (i.e., changes in the quantity or quality of muscle tissue, including fatty infiltration), several subsequent papers on this topic have reported conflicting findings [13–17]. Additional studies investigating specific clinical or activity-related features related to low back pain, including pain duration, disability, or pain intensity [18–22], physical function and muscle stiffness [16, 23, 24], physical demands of work [25], and radiculopathy-related outcomes [26–29], have also shown conflicting outcomes, failed to account for relevant confounders, or only had access to small to moderate population samples.

Therefore, the objective of this study was to explore the cross-sectional associations of lumbar multifidus muscle quality with pain, disability, spinal function, and work history among patients with non-specific low back pain and/or back-related leg pain. We hypothesized that greater multifidus quality would be associated with better clinical outcomes.

## Materials and methods

For this study, all patients presenting to the Spine Centre of Southern Denmark from September 2013 to October 2014 with a primary complaint of low back pain and/or lower extremity symptoms, who agreed to complete a comprehensive, prescribed electronic or paper-based clinical history questionnaire and undergo a standardized physical examination by a qualified clinician (i.e., a medical doctor, chiropractor, or physiotherapist), had their responses/results recorded in the SpineData registry [30]. From this pool, patients who also had lumbar MRIs available from the local hospital radiology department (acquired within one month of obtaining initial clinical data) were eligible for inclusion. Our initial exclusion criteria included patients with missing demographic information or a serious cause of low back pain (e.g., malignancy, infection, recent fracture). During the image selection process, cases were also excluded if they demonstrated at least one of the following: 1) failure to include the L4/5 or L5/S1 level; 2) any indication of surgery from L4 to S1; 3) no MRIs or T2-weighted axial images were provided; 4) duplicate cases; 5) poor image quality overall; 6) partial visualization or slice overlap artefact involving the lumbar multifidi at either level; and 7) distorted or unidentifiable L4 or L5 posterior arch anatomy. Other than surgery, patients’ prior treatment histories were not available for consideration within our selection criteria.

The clinical data from the SpineData registry were originally collected as part of a cohort study approved by the Danish Data Protection Agency for the Region of Southern Denmark (Journal number: 2008-58-0035-15/22513), performed following the Declaration of Helsinki principles, with written informed consent from all patients. Danish law does not require ethical approval from the Regional Committees on Health Research Ethics for Southern Denmark to access this data (a letter of exemption is available in Danish from the authors on request). Approval for analyzing this data within a larger project was provided by Murdoch University’s Human Research Ethics Committee (approval: 2017/110). A full description of the development and scope of the SpineData registry has been previously published [30].

### MRI acquisition and muscle measurement

Patient’s images were obtained using a body/spine coil on either a 1.0 T Philips Panorama (Best, The Netherlands) or 1.5 T Philips Achieva (Best, The Netherlands) MRI system. Axial T2-weighted turbo spin echo (TSE) sequences (angled along the individual L3/4 –L5/S1 disc planes) were used for muscle analysis, while sagittal T1-weighted TSE sequences were used to assist with slice level localization. On the T2-weighted TSE axial images, the lumbar multifidi were measured at L4/5 and L5/S1 bilaterally below the level of the exiting nerve roots. To help reduce the muscle outlining guesswork that can be encountered when using slices from a fixed location (e.g., the endplate), we selected the slice between the lower portion of the disc and the adjacent superior endplate which provided the fullest/clearest posterior arch anatomy and multifidus muscle outlines.

All lumbar multifidi measures were performed with sliceOmatic v5.0.8b [TomoVision, Magog, Canada]. Measurements were undertaken by an investigator with over 30 years of experience in spinal MRI interpretation, previous experience using sliceOmatic software in lumbar multifidus muscle analysis, and excellent reliability in performing the below-described measurement method [31]. The investigator was blinded to the patients’ demographic and outcome data prior to and during the measurement period. Muscle assessment focused on quality (i.e., pure muscle component) rather than quantity (i.e., total muscle area), as this may help provide a more nuanced muscle analysis and overcome shortcomings identified previously. For example, measures of total cross-sectional area fail to account for natural variations in overall lumbar multifidus size or to address proportionate changes between muscle and fat) [5, 21].

**Fig 1:**
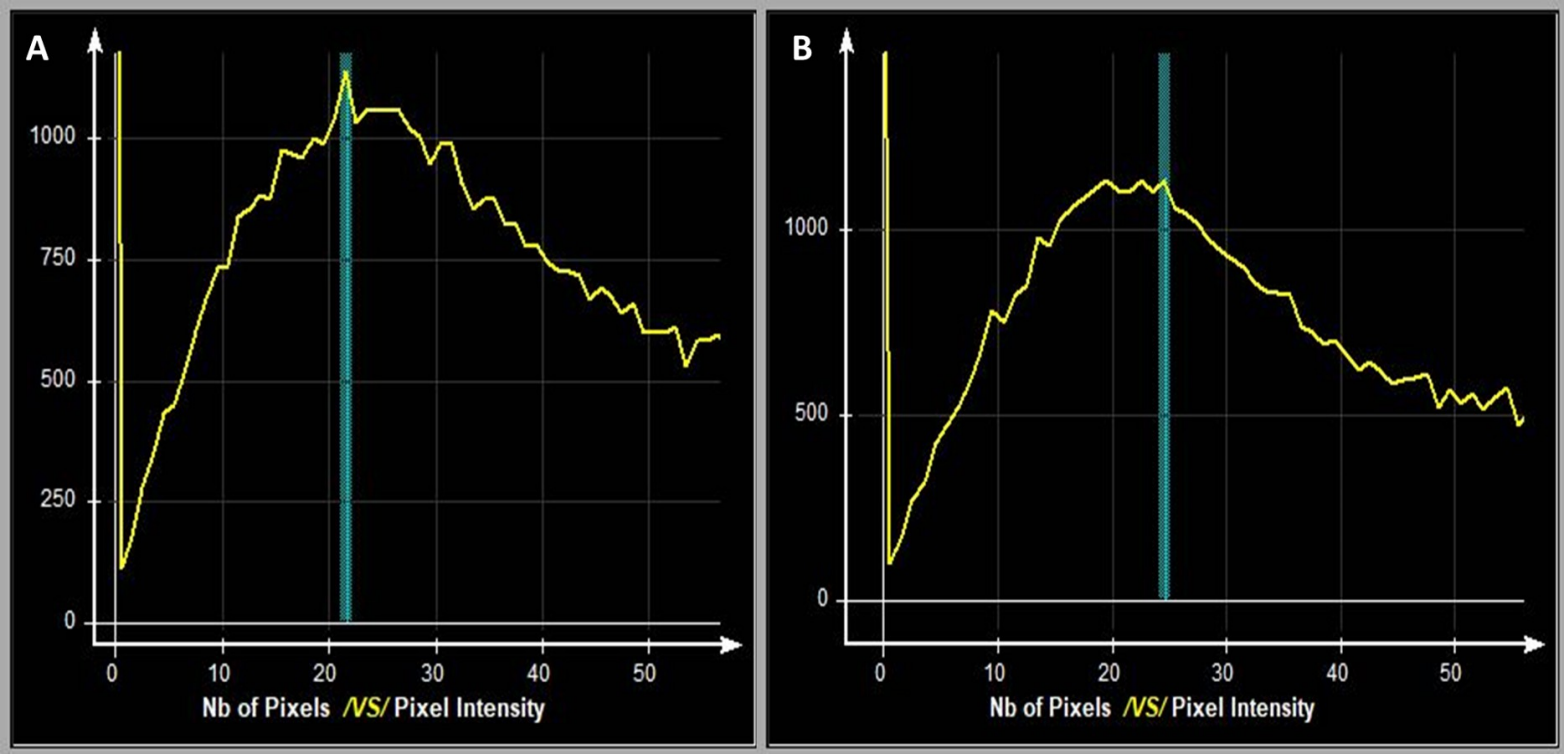
(A & B). Histogram examples of single (A) and triple (B) muscle peak values. Vertical blue lines intersect the histogram at the point of peak muscle signal intensity (i.e., 21 & 24 in these examples).

**Fig 2:**
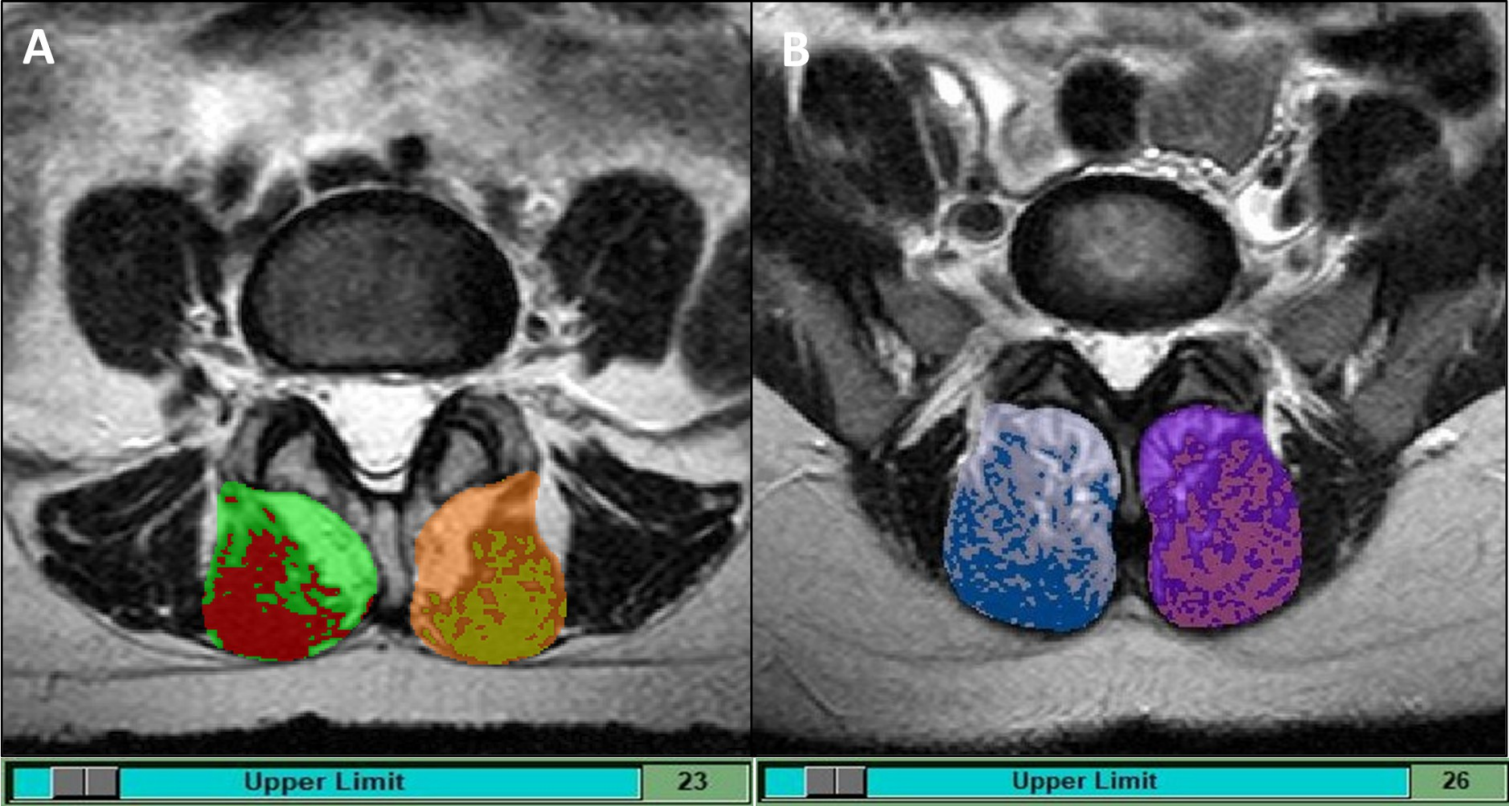
(A & B). Multifidus muscle total CSA outlines with peak muscle histogram values. Red and gold highlighting identifies the right and left muscle tissue areas contained within the peak value at L4 (A), while blue and pink highlights peak muscle at L5 (B).

To provide a reproducible estimate of pure muscle tissue, the maximum muscle signal intensity peak (single or multiple) within the image histogram (Fig 1A and 1B) was identified. This histogram represented the range of tissue signals across the image, with darker tissues (e.g., pure muscle) predominating at the lower end of the histogram scale. Next, to determine the total cross-sectional area and the percent of peak muscle cross-sectional area (% MCSA), the lumbar multifidi were outlined using the protocols developed for previous studies [5, 32]. This outline followed the posterolateral margins of the spinous process and lamina to the ipsilateral facet joint, the fascial separation between the multifidus and longissimus muscles, and the posterior fascial multifidus margin to the spinous process (Fig 2A and 2B). Finally, given the inclusion of patients with low back and/or leg-related outcomes in this study, we calculated the average % MCSA for all muscles at L4 and L5, and the lowest % MCSA for any muscle at L4 or L5 [peak muscle CSA (cm^2^) / total CSA (cm^2^) = % MCSA]. These muscle variables were selected to investigate whether the distribution of clinical findings affected the relationship between muscle quality and clinical outcomes differently (i.e., generalized clinical outcomes, such as back pain and disability (average % MCSA) versus focal outcomes, such as radicular pain (lowest % MCSA)). The full details of this lumbar multifidus muscle measurement protocol have been previously reported [31].

### Demographic information and clinical outcomes

We collected demographic information comprising age, sex, and self-reported height (cm) and weight (kg) for Body Mass Index (BMI) calculation. Clinical outcomes relating to pain characteristics, low back pain-related disability, occupational history, and physical examination findings were also included. The length of time since current low back or leg pain onset was categorized as: <3 months, 3–12 months, or >12 months). We used separate,11-point (0–10) numeric pain scales (NPS) to quantify low back and leg pain intensity, calculated as the average of: the current pain rating and the typical and worst pain ratings over the preceding 14 days [33]. Patients rated their level of low back pain-related disability using the 23-item version (0–23 scale) Roland Morris Disability Questionnaire (RMDQ) [34]. Low back morning stiffness was reported as: none, <30 minutes, 30–60 minutes, or > 60 minutes. Currently employed patients rated the physical demands of their work on a 10-point scale (1–10), with a higher rating indicating a more strenuous work environment. Although these last two variables are traditional rather than validated clinical measures, they were included in the development of the SpineData registry for use in clinical practice [30] and as such fit within our study parameters.

Physical examination outcomes included active lumbar range of motion and signs of lumbar radiculopathy. The clinician recorded the number of directions in which the patient indicated painful lumbar movement (flexion/extension, right/left rotation, right/left lateral flexion), and if they noted right or left-sided leg pain with signs of nerve root involvement (NRI) (defined as one or more of the following: a true positive straight-leg raise (SLR) test, or impaired deep tendon reflex, reduced muscle strength, or altered dermatome sensation of the painful extremity) [35].

### Statistical methods

Descriptive statistics for demographic, clinical, and physical examination variables were calculated. To investigate the associations between lumbar multifidus morphology and the clinical measures, we constructed separate univariable regression models, for each multifidus muscle measure (average and lowest % MCSA), per outcome. Model types depended on the nature of the dependent variables and their distributions. Linear models were used for normally distributed continuous outcomes (average low back pain rating; RMDQ score), Poisson models for count outcomes (total directions painful active range of motion (AROM)), Tobit models for censored outcomes with floor/ceiling effects (leg pain intensity), gamma models for negatively skewed outcomes (work rating), and multinomial logistic models for categorical outcomes (time since pain onset; morning stiffness; signs of NRI and leg pain). Residuals diagnostics were used to check model fit. Results were summarized according to model type with beta coefficients with standard errors, odds ratios (OR), or incidence rate ratios (IRR), and corresponding 95% confidence intervals. We also constructed adjusted models, accounting for age, sex, and BMI as potential confounders [36]. All regression outcomes are reported based on a 10% difference in muscle quality (e.g., 30% MCSA vs 40% MCSA; 55% MCSA vs 65% MCSA). All hypotheses were two-sided, and significance levels for all analyses were set at α = 0.05. All data were analyzed using Stata I/C version 17.0 (StataCorp, College Station, TX).

## Results

**Fig 3:**
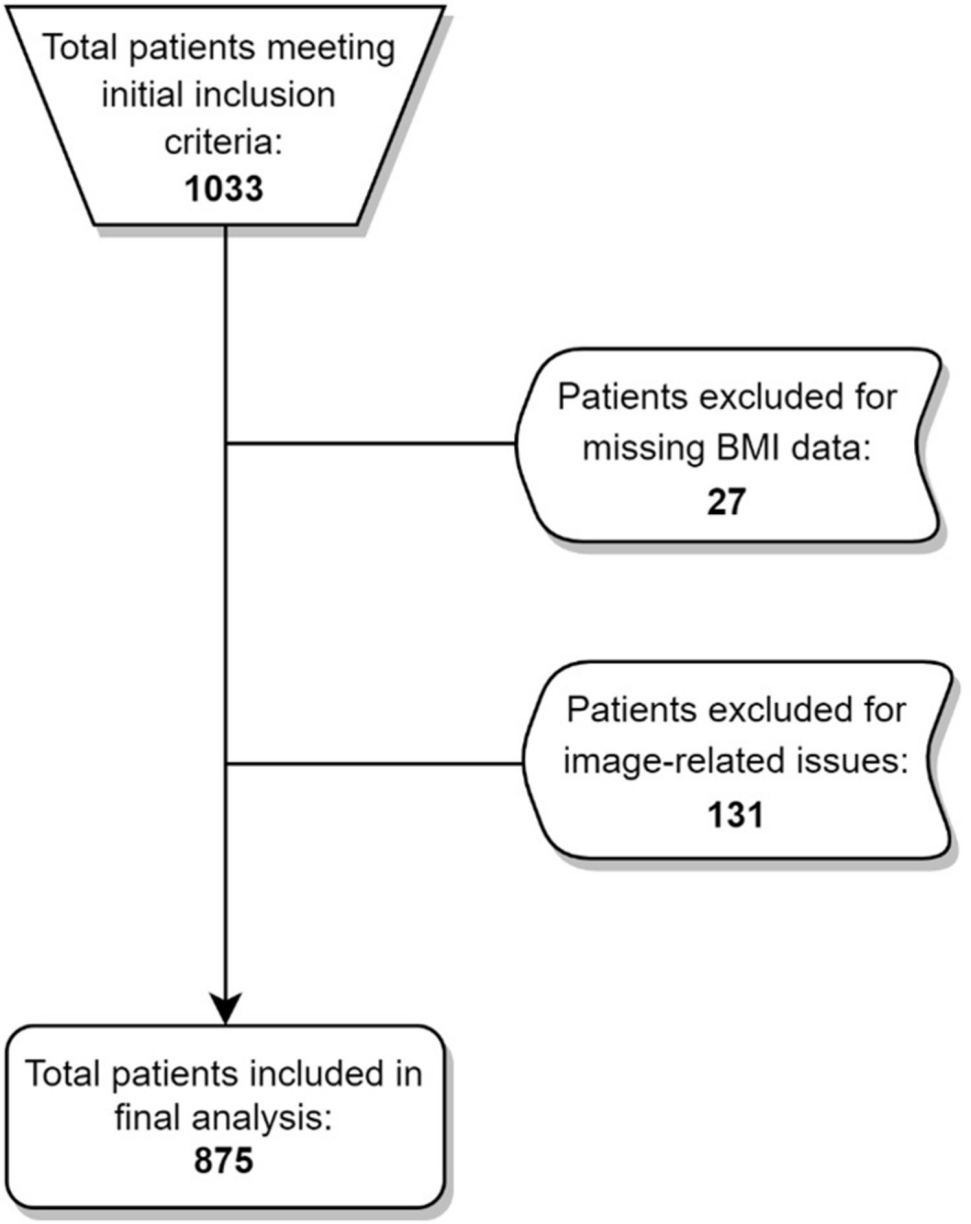
Case selection flowchart.

The patient selection process is presented in Fig 3. After excluding those with missing demographic data or for image-related reasons noted previously, 875 patients were eligible for inclusion in the final analysis. In some instances, either the patient or examining clinician did not provide input for a specific clinical variable, which resulted in a reduction of the number of evaluated cases for those variables. Additionally, only patients indicating current, active employment were included in the work rating analysis.

The final descriptive data are summarized in Table 1. The mean (SD) age was 43.6 (10.2); females comprised 55.7% of patients. Duration of current pain ranged from less than one month to over 40 years, with 2.3% of patients reporting no current low back pain and 13.4% reporting no current leg pain. All patients identified some level of low back disability, and the majority (63%) of employed patients indicated a less strenuous work history.

**Table 1:** Descriptive statistics for demographic, muscle, and clinical variables.

Demographic variables	N	
Age (years, at 1st visit)	875	43.6 (10.2)
BMI (kg/m^2^)	875	26.7 (4.8)
Female sex	875	487 (55.7%)
**Muscle variables**		
Average % MCSA	875	35.4 (11.9)
Lowest % MCSA	875	28.9 (11.8)
**Clinical variables**		
Low back pain intensity (0–10)	875	5.8 (2.2)
Leg pain intensity (0–10)	870	4.5 (2.9)
Low back pain-related disability (0–23)	864	13.1 (5.6)
Strenuous work history rating (1–10)	571	4.5 (2.8)
Symptom duration	875	
< 3 months		182 (21.0%)
3–12 months		323 (37.3%)
> 12 months		360 (41.6%)
Low back morning stiffness	871
None		204 (23.4%)
Present <30 minutes		216 (24.8%)
Present 30–60 minutes		209 (24.0%)
Present >60 minutes		242 (27.8%)
Painful lumbar AROM	690
No painful AROM		62 (9.0%)
Pain: 1 direction		152 (22.0%)
Pain: 2 directions		208 (30.1%)
Pain: 3 directions		104 (15.1%)
Pain: 4 directions		71 (10.3%)
Pain: 5 directions		30 (4.4%)
Pain: all directions		63 (9.1%)
Leg pain and NRI signs	769
No leg pain		117 (15.2%)
Leg pain with no signs of NRI		328 (42.7%)
Leg pain with signs of NRI		324 (42.1%)

Values are mean (SD) or counts (%). % MCSA = proportion of peak muscle cross-sectional area; AROM = active range of motion; NRI = nerve root involvement.

Model results are reported in Tables 2 and 3. Univariable models for the average % MCSA and lowest % MCSA demonstrated small but significant negative associations between lower muscle quality and greater leg pain intensity, low back disability, and likelihood of leg pain with signs of NRI. Conversely, higher muscle quality was associated with an increased risk of pain duration longer than 12 months. After adjusting for confounders, low back pain-related disability [β: -0.66, 95% CI: -1.05, -0.26] and pain onset duration over 12 months [OR: 1.28, 95% CI: 1.04, 1.55] retained their associations with lumbar multifidus quality for average % MCSA. Similarly for the lowest % MCSA, low back pain-related disability [β: -0.74, 95% CI: -1.14, -0.34], leg pain intensity [β: -0.25, 95% CI: -0.46, -0.03], and pain onset duration over 12 months [OR: 1.27, 95% CI: 1.03, 1.55] also remained associated with lumbar multifidus quality. There were no associations between lumbar multifidus quality and low back pain intensity, presence or length of morning stiffness, number of directions with painful lumbar AROM, or the physically strenuous nature of the patient’s work. Additional sensitivity analysis relating to the influence of symptom duration across all remaining clinical outcomes showed little or no change in effect size, with no change in the significance of any outcome measures.

**Table 2:** Associations between average % MCSA and outcomes.

		Univariable model			Adjusted model	
Outcome	N	Est.	95%CI	N	Est.	95%CI
Symptom duration^1^ <3 months	875	ref	ref	875	ref	ref
3 to 12 months		1.12	0.95, 1.29		1.10	0.90, 1.36
>12 months		**1.26**	**1.08, 1.47**		**1.28**	**1.04, 1.55**
Low back pain intensity	875	-0.03	-0.15, 0.09	875	-0.11	-0.27, 0.05
Leg pain intensity^2^	870	**-0.27**	**-0.43, -0.11**	870	-0.20	-0.41, 0.01
Low back disability	864	**-0.63**	**-0.94, -0.32**	864	**-0.66**	**-1.05, -0.26**
Morning stiffness^1^ None	871	ref	ref	871	ref	ref
<30 minutes		1.07	0.91, 1.27		1.03	0.83, 1.28
30 to 60 minutes		1.05	0.89, 1.23		1.06	0.86, 1.32
>60 minutes		1.00	0.86, 1.17		0.97	0.79, 1.20
Physically strenuous work rating^*3*^	571	0.00	-0.01, 0.01	571	0.01	0.00, 0.02
Directions with painful ROM (total)^4^	690	0.98	0.94, 1.02	690	0.99	0.94, 1.04
Leg pain and NRI signs^1^ No leg pain	769	ref	ref	769	ref	ref
Leg pain, no NRI		0.95	0.79, 1.02		0.99	0.94, 1.04
Leg pain with NRI		**0.78**	**0.65, 0.94**		0.83	0.66, 1.06

Results reported with unstandardized beta coefficients and 95% confidence intervals unless otherwise indicated. For outcomes, a linear regression model is used unless:

^1^ odds ratios from multinomial logistic model;

^2^ beta coefficients from Tobit model;

^3^ beta coefficients from gamma generalized linear model;

^4^ incident rate ratios from Poisson model. Adjusted models include sex, age, and body mass index. Est. = parameter estimates; CI = confidence interval; % MCSA = proportion of peak muscle cross-sectional area; ROM = range of motion; NRI = nerve root involvement. Results with a significant difference from the reference are **bolded**.

**Table 3:** Associations between lowest % MCSA and outcomes.

	Univariable model			Adjusted model		
Outcome	N	Est.	95%CI	N	Est.	95%CI
Symptom duration^1^ <3 months	875	ref	ref	875	ref	ref
3 to 12 months		1.14	0.97, 1.33		1.15	0.94, 1.41
>12 months		**1.26**	**1.07, 1.47**		**1.27**	**1.03, 1.55**
Low back pain intensity	875	-0.06	-0.19, 0.06	875	-0.15	-0.31, 0.00
Leg pain intensity^2^	870	**-0.30**	**-0.46, -0.14**	870	**-0.25**	**-0.46, -0.03**
Low back disability	864	**-0.69**	**-1.00, -0.38**	864	**-0.74**	**-1.14, -0.34**
Morning stiffness^1^ None	871	ref	ref	871	ref	ref
<30 minutes		1.06	0.90, 1.26		1.01	0.82, 1.26
30 to 60 minutes		1.04	0.89, 1.23		1.05	0.85, 1.31
>60 minutes		0.98	0.83, 1.15		0.92	0.75, 1.14
Physically strenuous work rating^*3*^	571	0.00	-0.01, 0.01	571	0.01	-0.01, 0.02
Directions with painful ROM (total)^4^	690	0.97	0.93, 1.01	690	0.99	0.93, 1.04
Leg pain and NRI signs^1^ No leg pain	769	ref	ref	769	ref	ref
Leg pain, no NRI		0.96	0.80, 1.14		1.01	0.97, 1.27
Leg pain with NRI		**0.80**	**0.67, 0.96**		0.87	0.69, 1.10

Results reported with unstandardized beta coefficients and 95% confidence intervals unless otherwise indicated. For outcomes, a linear regression model is used unless:

^1^ odds ratios from multinomial logistic model;

^2^ beta coefficients from Tobit model;

^3^ beta coefficients from gamma generalized linear model;

^4^ incident rate ratios from Poisson model. Adjusted models include sex, age, and body mass index. Est. = parameter estimates; CI = confidence interval; % MCSA = proportion of peak muscle cross-sectional area; ROM = range of motion; NRI = nerve root involvement. Results with a significant difference from the reference are **bolded**.

## Discussion

This study sought to explore the associations between lumbar multifidus muscle quality, and clinical outcomes comprising patient-reported clinical and occupational information, and physical examination findings. We found consistent associations between the % MCSA of the lumbar multifidi and low back-related disability and leg pain intensity, with lower disability and leg pain ratings in patients with a higher proportion of pure muscle CSA. This means that, on average, patients with higher multifidus quality reported lower levels of low back pain-related disability and leg pain intensity. However, each additional 10% of pure lumbar multifidus CSA was only associated with a 0.74-point lower RMDQ score, or a 0.25-point lower NRS leg pain score, as compared to reported minimal clinically important difference (MCID) values of at least 2.0 and 1.0 points, respectively, for the disability and pain instruments we used [34]. As such, the clinical importance or meaningfulness of these associations may be limited.

Our findings are consistent with results from several studies looking at low back disability using different measurement protocols, including different muscle measurements (total CSA of combined paraspinal muscles) [20], a composite pain/disability scale (Chronic Pain Grade Questionnaire) and muscle/fat grading system [22], and different imaging protocols (diagnostic ultrasound-measured lumbar multifidus CSA and thickness) [19]. Arguments highlighting the potential reasons for a relationship between altered lumbar multifidus size, structure, and/or function and reported low back disability appear plausible [19, 20]: the various causes of lumbar multifidus atrophy or reduced functionality may also contribute to or exacerbate existing lower lumbar spine instability and/or limit physical movement. This could result in perceived or real disability in this region and in turn contribute to further multifidus muscle atrophy. However, this explanation remains controversial as several small to moderate population sample studies have reported no relationships between altered paraspinal muscle morphology and low back disability [18, 21, 23, 37].

On the other hand, previously published small population sample studies assessing the relationships between altered lumbar multifidus morphology and leg pain intensity failed to identify any significant associations [38–40]. Therefore, the findings from this study appear to be the first to identify an inverse association that may exist between these two parameters. The most direct explanation for the limiting of this association to the lowest % MCSA category would be the presence of an underlying nerve root compromise which concurrently results in leg pain and isolated muscle quality reduction. While we did initially find a lower proportion of pure muscle was associated with a higher likelihood of patients presenting with leg pain and NRI, this association did not remain after adjusting for confounders. Although an association between altered lumbar multifidus muscle morphology and radiculopathy was noted in some studies [28, 29], no similar associations were identified in several other studies [26, 27, 40].

Barker et al. [18] identified a positive association between the duration of back pain and reduced lumbar multifidus quantity, whereas we noted a small, positive association between pain duration and increased lumbar multifidus quality. This appears to be a counterintuitive result, with no clear explanation as to why patients with higher paraspinal muscle quality would have a longer pain history. While muscle compensation from hyperactivity related to chronic pain is a possible explanation, this finding is more likely to be spurious than physiologic in nature. Symptom duration did not significantly affect any other outcome results. It should be noted that details regarding the onset of current symptoms (i.e., whether related to an initial onset versus being recurrent in nature) were not available. This may have influenced our outcomes when comparing muscle quality with pain duration.

The absence of association we noted between lumbar multifidus muscle quality and low back pain intensity was consistent with some studies [18, 20, 37], but not with others [19, 22]. Finally, we identified a lack of association between muscle quality and the physically strenuous nature of work, consistent with that reported by Fortin et al. [25]. We had postulated that reduced lumbar multifidus quality may limit a person’s ability to perform more physically demanding work, or that more time spent performing less demanding work may reduce multifidus muscle quality; however, our findings indicated such an association is unlikely to exist.

Several strengths and limitations of this study are acknowledged. We had access to a large patient population referred for low back and/or leg pain treatment, which allowed for more in-depth analysis of multiple clinical variables in a clinically relevant cohort. However, as all patients were symptomatic, comparison with a healthy population was not possible. Even though this study did identify some significant associations, they were relatively weak in scale. Additionally, the conflicting results between studies make true comparisons challenging. It is likely that some of these apparent conflicts are arising from the different populations being assessed, although the numerous variations in muscle measurement protocols are likely to be an additional contributing factor. To reduce the potential for selection bias and provide a more representative spectrum of low back or leg pain patient presentations across all age groups, we did not exclude patients with degenerative or other non-serious pathologies commonly found in a non-specific low back pain population. The presence of these co-morbidities had the potential to impact on the final outcomes; however, as the degree of impact is unknown, future cross-sectional and/or longitudinal studies could be directed at investigating any associations between combined clinical and pathological variables and the paraspinal muscles. Another potential limitation of this study was the lack of history regarding prior conservative treatment or exercise regimes–activities which may have confounded our results. But given that current muscle quality was being compared to the current clinical ratings, we anticipate prior conservative treatment will have had limited impact on our results. Further, the data collection process did not lend itself to identifying potential systemic neurological or myopathic disorders. However, patients in this study presented to the Spine Centre with a primary complaint related to the low back, which should essentially have excluded systemic neuromusculoskeletal conditions significant enough to affect our findings. As the MRIs in this study were acquired for clinical purposes, the slices were oriented along the disc planes at each level rather than being oriented perpendicular to the multifidus muscle. Some variation in muscle size/shape between patients may have occurred by selecting image slices that optimized outlining rather using a fixed slice location. However, using a narrow slice selection range and a proportion-based muscle analysis approach should have effectively negated the impact of any slice location variations. This study used several self-reported outcomes, relying on patient recall and interpretation. While validated NPS and RMDQ measures were implemented where possible [33, 34], other non-validated measures, such as time since onset of pain, timing of morning stiffness, and strenuous nature of work, were more subjective in nature. Lastly, the potential for brief time gaps between the acquisition of clinical measures and imaging could allow for some analysis error. As this would primarily be an issue in patients with acute pain, and only 1% of cases included acute pain patients with an imaging-to-outcome measure gap greater than one week, we anticipate these timing gaps would have minimally impacted the final results.

## Conclusion

The findings from this study support the hypothesis of an association between altered lumbar multifidus muscle morphology and clinical measures, specifically showing a greater proportion of pure multifidus muscle being associated with lower low back disability and leg pain intensity, but longer time since pain onset. The clinical importance of these findings is questionable, though, due to weak associations for all three outcomes. No associations were noted with the remaining clinical measures after adjusting for cofounders. As the presence of various co-morbidities may have impacted on the clinical measures, further investigation into the potentially complex interactions between clinical measures, spinal pathology, and multifidus muscle quality should be pursued to clarify the level of influence these may have on each other, and how this might relate to clinical outcomes or guide the management of patients with lumbar-related pain.

## Abbreviations

AROM: active range of motion
CSA: cross-sectional area
NPS: numeric pain scale
NRI: nerve root involvement
RMDQ: Roland Morris Disability Questionnaire
% MCSA: percent of peak muscle cross-sectional area

## References

[1] Vos T ea. Global burden of 369 diseases and injuries in 204 countries and territories, 1990–2019: A systematic analysis for the Global Burden of Disease Study 2019. Lancet. 2020;396(10258):1204–22. doi: 10.1016/S0140-6736(20)30925-9 33069326 PMC7567026

[2] Bardin LD, King P, Maher CG. Diagnostic triage for low back pain: A practical approach for primary care. Med J Aust. 2017;206(6):268–73. doi: 10.5694/mja16.00828 28359011

[3] Maher C, Underwood M, Buchbinder R. Non-specific low back pain. Lancet. 2017;389(10070):736–47. doi: 10.1016/S0140-6736(16)30970-9 27745712

[4] Deyo RA, Weinstein JN. Low back pain. N Engl J Med. 2001;344(5):363–70. doi: 10.1056/NEJM200102013440508 11172169

[5] Cooley JR, Walker BF, E MA, Kjaer P, Jensen TS, Hebert JJ. Relationships between paraspinal muscle morphology and neurocompressive conditions of the lumbar spine: A systematic review with meta-analysis. BMC Musculoskelet Disord. 2018;19(1):351. doi: 10.1186/s12891-018-2266-5 30261870 PMC6161433

[6] Ward SR, Kim CW, Eng CM, Gottschalk LI, Tomiya A, Garfin SR, et al. Architectural analysis and intraoperative measurements demonstrate the unique design of the multifidus muscle for lumbar spine stability. J Bone Jt Surg, Am Vol. 2009;91A(1):176–85. doi: 10.2106/JBJS.G.01311 19122093 PMC2663324

[7] Ward SR, Tomiya A, Regev GJ, Thacker BE, Benzl RC, Kim CW, et al. Passive mechanical properties of the lumbar multifidus muscle support its role as a stabilizer. J Biomech. 2009;42(10):1384–9. doi: 10.1016/j.jbiomech.2008.09.042 19457491 PMC2752430

[8] Wilke HJ, Wolf S, Claes LE, Arand M, Wiesend A. Stability increase of the lumbar spine with different muscle groups. A biomechanical in vitro study. Spine. 1995;20(2):192–8. 7716624 10.1097/00007632-199501150-00011

[9] Freeman MD, Woodham MA, Woodham AW. The role of the lumbar multifidus in chronic low back pain: A review. PM&R. 2010;2(2):142–6. doi: 10.1016/j.pmrj.2009.11.006 20193941

[10] Frymoyer JW, Selby DK. Segmental instability. Rationale for treatment. Spine. 1985;10(3):280–6. doi: 10.1097/00007632-198504000-00017 3992349

[11] Shaughnessy M, Caulfield B. A pilot study to investigate the effect of lumbar stabilisation exercise training on functional ability and quality of life in patients with chronic low back pain. Int J Rehabil Res. 2004;27(4):297–301. doi: 10.1097/00004356-200412000-00007 15572993

[12] Fortin M, Macedo LG. Multifidus and paraspinal muscle group cross-sectional areas of patients with low back pain and control patients: A systematic review with a focus on blinding. Phys Ther. 2013;93(7):873–88. doi: 10.2522/ptj.20120457 23504343 PMC3704232

[13] Cuellar WA, Wilson A, Blizzard CL, Otahal P, Callisaya ML, Jones G, et al. The assessment of abdominal and multifidus muscles and their role in physical function in older adults: A systematic review. Physiotherapy. 2017;103(1):21–39. doi: 10.1016/j.physio.2016.06.001 27667760

[14] Dahlqvist JR, Vissing CR, Hedermann G, Thomsen C, Vissing J. Fat replacement of paraspinal muscles with aging in healthy adults. Med Sci Sports Exerc. 2017;49(3):595–601. doi: 10.1249/MSS.0000000000001119 27741218

[15] Goubert D, van Oosterwijck J, Meeus M, Danneels L. Structural changes of lumbar muscles in non-specific low back pain. Pain Physician. 2016;19(7):E985–E1000. 27676689

[16] Masaki M, Aoyama T, Murakami T, Yanase K, Ji X, Tateuchi H, et al. Association of low back pain with muscle stiffness and muscle mass of the lumbar back muscles, and sagittal spinal alignment in young and middle-aged medical workers. Clin Biomech (Bristol, Avon). 2017;49:128–33. doi: 10.1016/j.clinbiomech.2017.09.008 28934633

[17] Ogon I, Takebayashi T, Takashima H, Morita T, Yoshimoto M, Terashima Y, et al. Quantitative analysis concerning atrophy and fat infiltration of the multifidus muscle with magnetic resonance spectroscopy in chronic low back pain. Spine Surg Relat Res. 2019;3(2):163–70. doi: 10.22603/ssrr.2018-0023 31435570 PMC6690088

[18] Barker KL, Shamley DR, Jackson D. Changes in the cross-sectional area of multifidus and psoas in patients with unilateral back pain: The relationship to pain and disability. Spine. 2004;29(22):E515–9. doi: 10.1097/01.brs.0000144405.11661.eb 15543053

[19] Emami F, Yoosefinejad AK, Razeghi M. Correlations between core muscle geometry, pain intensity, functional disability and postural balance in patients with nonspecific mechanical low back pain. Med Eng Phys. 2018;60:39–46. doi: 10.1016/j.medengphy.2018.07.006 30077486

[20] Ranger TA, Cicuttini FM, Jensen TS, Heritier S, Urquhart DM. Paraspinal muscle cross-sectional area predicts low back disability but not pain intensity. Spine J. 2019;19(5):862–8. doi: 10.1016/j.spinee.2018.12.004 30529786

[21] Rezazadeh F, Taheri N, Okhravi SM, Hosseini SM. The relationship between cross-sectional area of multifidus muscle and disability index in patients with chronic non-specific low back pain. Med Eng Phys. 2019;42:1–5. doi: 10.1016/j.msksp.2019.03.005 30981101

[22] Teichtahl AJ, Urquhart DM, Wang Y, Wluka AE, Wijethilake P, O’Sullivan R, et al. Fat infiltration of paraspinal muscles is associated with low back pain, disability, and structural abnormalities in community-based adults. Spine J. 2015;15(7):1593–601. doi: 10.1016/j.spinee.2015.03.039 25828477

[23] Hildebrandt M, Fankhauser G, Meichtry A, Luomajoki H. Correlation between lumbar dysfunction and fat infiltration in lumbar multifidus muscles in patients with low back pain. BMC Musculoskelet Disord. 2017;18(1):12. doi: 10.1186/s12891-016-1376-1 28068962 PMC5223418

[24] Sions JM, Coyle PC, Velasco TO, Elliott JM, Hicks GE. Multifidi muscle characteristics and physical function among older adults with and without chronic low back pain. Arch Phys Med Rehabil. 2017;98(1):51–7. doi: 10.1016/j.apmr.2016.07.027 27590444 PMC5183461

[25] Fortin M, Videman T, Gibbons LE, Battié MC. Paraspinal muscle morphology and composition: A 15-yr longitudinal magnetic resonance imaging study. Med Sci Sports Exerc. 2014;46(5):893–901. doi: 10.1249/MSS.0000000000000179 24091994

[26] Farshad M, Gerber C, Farshad-Amacker NA, Dietrich TJ, Laufer-Molnar V, Min K. Asymmetry of the multifidus muscle in lumbar radicular nerve compression. Skeletal Radiol. 2014;43(1):49–53. doi: 10.1007/s00256-013-1748-7 24170037

[27] Frost LR, Brown SH. Neuromuscular ultrasound imaging in low back pain patients with radiculopathy. Man Ther. 2016;21:83–8. 26037592 10.1016/j.math.2015.05.003

[28] Hyun JK, Lee JY, Lee SJ, Jeon JY. Asymmetric atrophy of multifidus muscle in patients with unilateral lumbosacral radiculopathy. Spine. 2007;32(21):E598–602. doi: 10.1097/BRS.0b013e318155837b 17906560

[29] Min JH, Choi HS, Ihl Rhee W, Lee JI. Association between radiculopathy and lumbar multifidus atrophy in magnetic resonance imaging. J Back Musculoskelet Rehabil. 2013;26(2):175–81. doi: 10.3233/BMR-130365 23640319

[30] Kent P, Kongsted A, Jensen TS, Albert HB, Schiøttz-Christensen B, Manniche C. Spinedata - a Danish clinical registry of people with chronic back pain. Clin Epidemiol. 2015;7:369–80. doi: 10.2147/CLEP.S83830 26316820 PMC4540159

[31] Cooley JR, Jensen TS, Kjaer P, Jacques A, Theroux J, Herbert JJ. Spinal degeneration is associated with lumbar multifidus morphology in secondary care patients with low back or leg pain. Sci Rep. 2022;12:14676. doi: 10.1038/s41598-022-18984-1 36038653 PMC9424282

[32] Cooley JR, Hebert JJ, de Zoete A, Jensen TS, Algra PR, Kjaer P, et al. Assessing lumbar paraspinal muscle cross-sectional area and fat composition with T1 versus T2-weighted magnetic resonance imaging: Reliability and concurrent validity. PLoS One. 2021;16(2):e0244633. doi: 10.1371/journal.pone.0244633 33544707 PMC7864460

[33] Manniche C, Asmussen K, Lauritsen B, Vinterberg H, Kreiner S, Jordan A. Low back pain rating scale: Validation of a tool for assessment of low back pain. Pain. 1994;57(3):317–26. doi: 10.1016/0304-3959(94)90007-8 7936710

[34] Lauridsen HH, Hartvigsen J, Manniche C, Korsholm L, Grunnet-Nilsson N. Responsiveness and minimal clinically important difference for pain and disability instruments in low back pain patients. BMC Musculoskelet Disord. 2006;7:82. doi: 10.1186/1471-2474-7-82 17064410 PMC1635558

[35] Kongsted A, Kent P, Albert H, Jensen TS, Manniche C. Patients with low back pain differ from those who also have leg pain or signs of nerve root involvement - a cross-sectional study. BMC Musculoskelet Disord. 2012;13:236. doi: 10.1186/1471-2474-13-236 23190800 PMC3585913

[36] Ranger TA, Cicuttini FM, Jensen TS, Peiris WL, Hussain SM, Fairley J, et al. Are the size and composition of the paraspinal muscles associated with low back pain? A systematic review. Spine J. 2017;17(11):1729–48. doi: 10.1016/j.spinee.2017.07.002 28756299

[37] Cankurtaran D, Yigman ZA, Umay E. Factors associated with paravertebral muscle cross-sectional area in patients with chronic low back pain. Korean J Pain. 2021;34(4):454–62. doi: 10.3344/kjp.2021.34.4.454 34593663 PMC8494955

[38] Fortin M, Lazary A, Varga PP, Battie MC. Association between paraspinal muscle morphology, clinical symptoms and functional status in patients with lumbar spinal stenosis. Eur Spine J. 2017, doi: 10.1007/s00586-017-5228-y 28748488

[39] Miki T, Naoki F, Takashima H, Takebayashi T. Associations between paraspinal muscle morphology, disc degeneration, and clinical features in patients with lumbar spinal stenosis. Prog Rehabil Med. 2020;5:20200015. doi: 10.2490/prm.20200015 32844128 PMC7429555

[40] Kader DF, Wardlaw D, Smith FW. Correlation between the MRI changes in the lumbar multifidus muscles and leg pain. Clin Radiol. 2000;55(2):145–9. doi: 10.1053/crad.1999.0340 10657162

